# Nurses’ knowledge of chemotherapy-induced neutropenia and its management: a cross-sectional survey

**DOI:** 10.1007/s00432-022-04140-9

**Published:** 2022-07-12

**Authors:** Mohammad Al Qadire, Cherry Ann C. Ballad, Ma’en Aljezawi, Omar Al Omari, Fawwaz Alaloul, Ahmad Musa, Sulaiman Al Sabei, Atika Khalaf

**Affiliations:** 1 College of Nursing, Sultan Qaboos University, PC 123, P.O. Box 66, Muscat, Sultanate of Oman; 2 Faculty of Nursing, Al Al-Bayt University, P.O. Box 130040, Mafraq, 25113 Jordan; 3 Faculty of Health Sciences, Kristianstad University, Elmetorpsvägen 15, 291 88 Kristianstad, SE Sweden

**Keywords:** Cancer, Chemotherapy-induced neutropenia, Neutropenia, Nurses, Oncology nursing

## Abstract

**Background:**

Chemotherapy-induced neutropenia (CIN) is a serious and potentially life-threatening condition that is associated with high morbidity, mortality, and healthcare costs.

**Objective:**

This study aims to assess nurses’ level of knowledge of CIN and its association with socio-demographic factors.

**Methods:**

A cross-sectional survey design was used. Results: Participants had a mean age of 34.1 years (SD = 7.1 years) and were predominantly female (78%) and with a bachelor’s degree in nursing (95.6%). The nurses had a moderate level of knowledge about neutropenia and its management (mean total score 16.3 out of 30, SD = 3.7). Those who had a post-graduate degree (*P* = .048), had received an oncology educational course (*P* = .011), had attended a course on neutropenia (*P* = .007), who were working in an oncology unit (*P* = .002), and had more oncology experience (*P* = 001) were more likely to have a higher level of knowledge of CIN and its management compared to their other counterparts.

**Conclusion:**

Based on the findings of a moderate level of knowledge of CIN among nurses, the findings call for the need for further education and training. As a long-term plan, this might be accomplished by encouraging nurses to pursue post-graduate education or oncology-specialized certification and supporting them with scholarship grants. However, deliberate plans for short courses, training and workshops on oncology or CIN are other choices with a more immediate impact on nurses’ knowledge and clinical practice. Finally, integrating oncology nursing education within nursing curricula is urgently needed.

## Introduction

Cancer is a significant health and economic burden affecting more than 50 million people worldwide, with over 19 million new cases discovered in 2020 (The Global Cancer Observatory 2020). It is the second leading cause of death globally, accounting for nearly 10 million deaths a year (The Global Cancer Observatory 2020). In Oman, 21,000 cancer cases were recorded among the Omani population in the period 1996–2015, an average of 1,050 cases annually (Al-Lawati et al. 2019). Chemotherapy is one of the gold standards in cancer treatment (Schelenz et al. 2012). However, its use has a considerable number of side effects, including hematopoietic suppression which results in neutropenia, known as chemotherapy-induced neutropenia (CIN) (Crawford et al. 2004; Sureda et al. 2019).

Neutropenia is serious and potentially life threatening; it occurs when neutrophil is not produced at a desirable rate, dulling the inflammatory response and predisposing the individual to a higher risk of the development of infection (Yarbro et al. 2018). Clinically, neutropenia is defined as having an absolute neutrophil count (ANC) less than 1500/mm^3^; it often requires hospitalization and belligerent treatment to prevent sepsis (Yarbro et al. 2018). CIN is associated with higher morbidity, mortality and healthcare costs (Abou Saleh et al. 2013; Crawford et al. 2004; Lyman et al. 2010; Schelenz et al. 2012). Once neutropenia is detected, chemotherapeutic treatments are reduced or withheld, which further compromises survival and negatively affects the course of treatment (Crawford et al. 2004; Schelenz et al. 2012; Sureda et al. 2019).

Oncology nurses have a fundamental role in easing the burden of patients suffering from the consequences of neutropenia. They must have the knowledge, proficient skills, and a compassionate attitude to ensure optimal care and reduce patient suffering (Kvåle and Bondevik 2010). However, nurses in other units such as medical, surgical, emergency, and intensive care also deal with cancer patients. As patient advocates, nurses can put their knowledge to good use by working collaboratively with the multidisciplinary team and recommending evidence-based interventions to the advantage of their patients (Kaplow and Spinks 2015). For example, early prophylactic treatments have been found to curtail hospital stay (Gerlier et al. 2010). While these are essential features, knowledge of CIN among nurses is a topic largely unexplored in nursing research and scholarship, and the literature is inconclusive when it comes to related concepts such as nurses’ knowledge and practice of infection control and prevention.

### Literature review

A comprehensive literature review reveals a dearth of studies assessing the knowledge of oncology nurses of CIN and its management (Naghdi et al. 2019; Nirenberg et al. 2010; Tarakcioglu Celik and Korkmaz 2017; Teleb Osman and Mohamed Bayoumy 2016). In addition, these studies provide contrasting findings.

Two studies, in Turkey and America, show that oncology nurses have a generally high level of knowledge of CIN and neutropenic patient management (Nirenberg et al. 2010; Tarakcioglu Celik and Korkmaz 2017). More specifically, they have a high level of knowledge of neutrophil functions, clinical manifestations of infection and suitable nursing care in neutropenic patients, although poor adherence to infection control practices such as hand hygiene and preparation and administration of medicines (Tarakcioglu Celik and Korkmaz 2017). On the other hand, a study conducted in Iran shows that nurses had moderate knowledge of CIN and moderate practice of infection control and prevention (Naghdi et al. 2019). It is noteworthy that very few nurses have commendable practice (< 20%). There is also a significant correlation between their knowledge and practice for vital signs assessment and medication preparation (Naghdi et al. 2019). These studies indicate that nurses’ knowledge and practice in the care of patients with CIN are suboptimal, with a clear knowledge-practice gap.

It is not surprising for nurses to encounter patients with cancer in non-cancer units such as medical or surgical units. Because the infection is one of the significant life-threatening complications of CIN, nurses must have adequate knowledge and practice of infection control and prevention. Many studies indicate that nurses’ general knowledge of infection control is high (Chuc et al. 2018; Gulilat and Tiruneh 2014; Okanlawon 2014; Parmeggiani et al. 2010; Suliman et al. 2018). However, most reflect that nurses’ practice of infection prevention is average (Sarani et al. 2016) or poor (Chuc et al. 2018; Gulilat and Tiruneh 2014; Parmeggiani et al. 2010). On a very alarming note, many studies indicated the wide knowledge-practice gap with regard to infection control practices (Accardi et al. 2017; Adegboye et al. 2018; Chuc et al. 2018; Gulilat and Tiruneh 2014; Nasiri et al. 2019; Okanlawon 2014; Suliman et al. 2018; Tenna et al. 2013) which clearly calls for immediate corrective action.

With the goal of improving nurses’ knowledge of CIN and practice in the care of neutropenic patients, a quasi-experimental study was conducted to evaluate the impact of a nursing intervention bundle on the prevention of neutropenia-associated infections (Teleb Osman and Mohamed Bayoumy 2016). The result for the baseline data collection point indicated that nurses had poor knowledge of neutropenia and preventive measures against infection. Their knowledge significantly improved after they were subjected to an intensive educational program (Teleb Osman and Mohamed Bayoumy 2016). However, the use of a non-randomized non-controlled design, convenience sampling and small sample size (*n* = 30) makes it difficult to generalize the results of the study. It is also important to note that the nurses’ knowledge was somehow reduced two months after the intervention (Teleb Osman and Mohamed Bayoumy 2016).

An extensive literature search revealed that nurses’ knowledge of CIN and corresponding patient care is an unexplored topic in the Sultanate of Oman. Such a study could provide vital information for nursing administrators, nurses, clinical educators and nursing scholars alike. The findings could be utilized as a baseline for the implementation of nursing education programs and policy development. As the healthcare professionals who spend the most time with the patient, nurses play a crucial role in preventing the occurrence of infection or its likely dangerous progression at the earliest point possible. Nurses are at the forefront in identifying patients at risk of infection, and possible sources of infection, caring and educating patients with CIN, monitoring symptoms, carrying out effective infection control strategies and taking action at the very first sign of CIN and infection (Kaplow and Spinks 2015). Being equipped with the right knowledge is fundamental to establish the quality of care. Hence, this study aims to assess the nurses’ level of knowledge of CIN and its management, and the association of knowledge with their socio-demographic factors.

## Methods

### Design

This study utilized a cross-sectional survey design.

### Sample, sampling, and sample size

The study sample consisted of 182 nurses who were practicing in oncology units. Nurses working in medical and surgical units, and intensive care units, were also included as they occasionally receive patients with cancer. All nurses who have a bachelor’s degree in nursing or above, have working experience of at least six month, and have agreed to participate, were included. The research team utilized a simple random sampling technique. A list of all nurses was obtained and numbered sequentially. Then, a computer-generated list of 181 numbers was constructed, and those with selected numbers were contacted.

The sample size depended on the possible proportion of correct responses in the neutropenia knowledge assessment tool. For 50% correct answers, knowing that there are about 340 nurses meeting the inclusion criteria, a sample size of 181 is considered adequate. From http://www.raosoft.com/samplesize.html, this would allow the percentage of correct answers to be estimated with a 95% margin-of-error of at most ± 5%.

### Settings

The study was conducted in a large referral hospital where most cancer patients are treated. This hospital is located in the capital city Muscat, and it has oncology pediatric and adult inpatient wards. It has outpatient chemotherapy clinics and a bone marrow transplant unit.

### Instruments

A demographic data sheet and neutropenia knowledge questionnaire were used to collect the required data.

*Demographic data sheet* collected information about nurses’ age, gender, education level, years of experience, working unit, nationality and previous education about oncology nursing and neutropenia.

*Neutropenia Knowledge Questionnaire* is a tool to evaluate nurses’ knowledge of neutropenia and the care of neutropenic patients (Tarakcioglu Celik and Korkmaz 2017). It comprises 30 true/false statements with an “I don’t know” option to avoid guessing. Each correct answer is given a score of “1”, otherwise zero. The responses to all items are summed to produce the total score. A score range of 0–10 indicates a poor level of knowledge, 11–20 moderate knowledge and 21–30 good knowledge (Naghdi et al. 2019; Tarakcioglu Celik and Korkmaz 2017). The tool is reported to have established content validity (content validity index 0.95) and reliability (Cronbach alpha = 0.7) (Naghdi et al. 2019). The English version of the tool was used.

### Ethical considerations

The required ethical approvals were obtained from the College of Nursing Ethics Committee (Ref. No. CON/NF/34) and Medical Ethics Committee (Ref. No. SQU-EC/331/2021) prior to embarking on the study. All participants were given information about the study’s purpose and requirements. This information was provided in the first section of the questionnaire. All participants were informed that their participation was voluntary, no names or identifying data would be collected, and that completing the questionnaire was considered implicit consent. No harm or risk was expected because of participation in this study.

### Data collection procedure

Following the required ethical approval, the head nurses of the respective units were approached to explain the study’s purposes and procedure. Then, a list of nurses and their phone numbers from each unit was obtained. They were compiled into a single list and numbered sequentially. Using available free online resources, a list of 181 randomly selected numbers was generated. Then, an invitation to participate was sent to potential respondents; if they showed interest in participating in the study, a member of the research team called them and arranged a meeting. During this meeting, conducted in the workplace, nurses completed the questionnaire and returned it to the research team either in person or by putting in the designated box within the unit.

### Data analysis

All questionnaires were first assessed for completeness, then coded and entered into SPSS version 23. Descriptive statistics such as means, frequencies and percentage were used to describe the sample’s characteristics and knowledge level. In addition, independent *t* test, ANOVA and Pearson correlation tests were used to analyze the association between the nurses’ knowledge level and the socio-demographic variables.

## Results

### Nurses’ characteristics

The total number of nurses who agreed to participate and completed the study was 182. Participants’ characteristics are detailed in Table 1. Their mean age was 34.1 years (SD = 7.1 years), with females being dominant in the sample (78%). Most (95.6%) had a bachelor’s degree in nursing. Participants were divided almost equally among three working areas: oncology units (32.4%), medical and surgical units (32.4%), and intensive care units (35.2%). Most of the nurses were of Indian (44%) or Omani nationality (40.1%), with 15.9% being Filipino. The majority of the sample had not received any educational program either on oncology nursing (74.2%) or on neutropenia (86.3%)).

**Table 1:** Demographic characteristics of participants (*n* = 182)

Characteristic	Frequency (%)	Mean (SD)
Age (year)		34.1 (7.1)
Nursing experience (year)		9.6 (7.2)
Oncology nursing experience (year)		2.3 (4.2)
*Gender*		
Male	40 (22.0)	
Female	142 (78.0)	
*Education level*		
Bachelors	174 (95.6)	
Postgraduate (MSN/PhD)	8 (4.4)	
*Working area*		
Oncology units	59 (32.4)	
Medical and surgical units	59 (32.4)	
Intensive care units	64 (35.2)	
*Nationality*		
Indian	80 (44.0)	
Omani	73 (40.1)	
Filipino	29 (15.9)	
*Have you received oncology nursing education course*?		
Yes	47 (25.8)	
No	135 (74.2)	
*Have you received education course about Neutropenia*?		
Yes	25 (13.7)	
No	157 (86.3)	

### Nurses’ Knowledge of Neutropenia and its Management

The total sample of nurses had a mean score of 16.3 out of 30 (SD = 3.7) on the Neutropenia Knowledge Questionnaire, corresponding to moderate knowledge of neutropenia. Some 16 questions were answered correctly by 50% or more of the nurses. Table 2 presents the frequency of the correct answer for each item in the Knowledge Questionnaire. Here it can be noticed that the top three correctly answered questions were: item number 30 (95.6%), which asked about informing patients and family about the infection control procedure; item number 24 (91.8%), which asked if neutropenic patients must be put in a private room; and item number 29 (88.5%), which asked if the skin and mucosal membranes should be assessed on a daily basis. Conversely, the three least correctly answered questions were: item number 19 (6.6%), which asked about rinsing a neutropenic patient’s mouth three times a day; item number 11 (10.4%), which asked about bathing the neutropenic patients on a daily basis; and item number 27 (18.7%), which asked about wearing gloves, masks, and gowns during neutropenic patients’ care. It is also worth mentioning here that less than half of the nurses (48.4%) knew the correct definition of neutropenia, represented by item number 3 in the questionnaire. Overall, nurses mainly lacked the knowledge related to the detention and assessment of neutropenia, infection control standards and aspects of its management.

**Table 2:** Students Nurses Knowledge of Neutropenia Management (*n* = 182)

Itemnno	Item	Correct Responses	
		*n*	%
1	Neutropenia is characterized by a decrease in neutrophils and thrombocytes. (False*)	44	24.2
2	Neutrophils provide the body’s defence by phagocytosis of microorganisms. (True*)	154	84.6
3	A patient is classified as neutropenic when the neutrophil count is 2500 cells/mm3. (False*)	88	48.4
4	Lymphoma” is one of the diseases that cause neutropenia. (True*)	136	74.7
5	Hypotension, an indicator for sepsis, is an important symptom for neutropenic patients. (True*)	158	86.8
6	If the general condition of patient with neutropenia is stable, his or her vital signs can be assessed every 8 h. (False*)	75	41.2
7	It is difficult to identify the signs and symptoms of infection in patients with neutropenia. (True*)	56	30.8
8	Radiotherapy leads to neutropenia by suppressing bone marrow function. (True*)	135	74.2
9	Patients’ own flora is the most likely source of microorganisms in neutropenic patients. (True*)	110	60.4
10	IV catheter dressings should be changed every 4 h in neutropenic patients. (False*)	137	75.3
11	Neutropenic patients should take a shower/bath or be given a bed bath daily. (False*)	19	10.4
12	IV injections should be preferred rather than subcutaneous or intramuscular injections. (True*)	104	57.1
13	Neutropenic patients should avoid coughing and deep breathing exercises. (False*)	134	57.1
14	One of the signs of infection in patients with neutropenia is glycosuria. (True*)	38	20.9
15	Enemas can be used in neutropenic patients’ constipation. (False*)	92	50.5
16	Gastrointestinal system infections likely develop frequently in neutropenic patients. (True*)	144	79.1
17	Stomatitis may occur often in neutropenic patients’ oral mucosa. (True*)	150	82.4
18	Neutropenic patients’ oral care is provided with sodium bicarbonate solution. (True*)	63	34.6
19	Neutropenic patients’ oral care includes rinse of mouth three times a day. (False*)	12	6.6
20	Drinking tap water is not recommended for neutropenic patients. (True*)	129	70.9
21	Neutropenic patients’ diet includes plenty of fresh vegetables and fruits to meet vitamins needs. (False*)	74	40.7
22	Respiratory isolation is used with neutropenic patients. (False*)	52	28.6
23	Nobody should get in neutropenic patient’s room except the health care providers. (False*)	45	24.7
24	Neutropenic patients must be placed in private rooms. (True*)	167	91.8
25	Neutropenic patient rooms should be cleaned after other ward areas by the cleaning staff. (False*)	84	46.2
26	Floor must be cleaned with a damp mop. (True*)	86	47.3
27	During patient care, gowns, masks, and gloves should be worn. (False*)	34	18.7
28	Urinary catheterization must be performed to measure the urine output. (False*)	118	64.8
29	Skin and mucous membranes should be assessed daily and documented. (True*)	161	88.5
30	Nurses should inform patient and family about infection control procedures. (True*)	174	95.6

^*^The correct answer

### Comparison of total neutropenia knowledge scores

To check the distribution of the total mean knowledge score between the different study groups identified in Table 1, an independent t-test was used for groups with two categories, i.e., gender, education level, receiving education about oncology nursing or management of neutropenia; a one-way ANOVA was used for groups with more than two categories, i.e., nationality and working area.

The results of the t test showed that: nurses who had a post-graduate degree had a significantly higher knowledge score than those with a bachelor’s degree (*P* = 0.048); nurses who had received an oncology educational course had a significantly higher knowledge score than those who had not (*P* = 0.011); nurses who had received an education course about neutropenia had a significantly higher knowledge score than those who had not (*P* = 0.007). However, there was no statistical difference in the total knowledge scores between male nurses and female nurses (P = 0.265). The results of the comparative analysis are presented in Table 3. Further, the one-way ANOVA analysis showed that nurses who were working in the oncology unit had a significantly higher knowledge score than those working in other units (*P* = 0.002); and Filipino nurses had a significantly higher knowledge score compared to other nationalities (*P* < 0.001) (Table 3).

**Table 3:** Comparisons of total knowledge score between nurses’ characteristics (*n* = 182)

Characteristic	Mean (SD)	df	*P*-value	95% CI
*Gender*^#^		180	.265	– 0.57 to 2.07
Male	15.8 (2.9)	(223.6)		
Female	16.5 (3.9)			
*Education level*^#^		180	.048*	– 3.72 to – 0.24
Bachelors	16.2 (3.7)			
Postgraduate (MSN/PhD)	18.1 (2.1)			
Have you received oncology nursing education course?		180	.011*	– 2.48 to – 0.33
Yes	17.4 (2.1)			
No	15.9 (3.9)			
Have you received education course about Neutropenia?		180	0.007*	– 2.09 to – 0.55
Yes	17.5 (2.9)			
No	15.7 (3.9)			
Working area ^¤^		179	.002	
Oncology units	17.5 (2.8)			
Medical and surgical units	16.5 (4.4)			
Intensive care units	15.1 (3.5)			
Nationality ^¤^		179	< .001	
Indian	17.3 (2.8)			
Omani	14.8 (4.4)			
Filipino	17.4 (2.7)			

^*^Significant at *p* ≤ .05, ^#^*t*-test, ANOVA

To further understand factors affecting total knowledge score, a Pearson correlation test was used to measure if there was any correlation between total knowledge score and age, years of experience, and oncology experience. Results of the correlation showed that there was a statistically significant weak positive correlation between total score and age, *r* (182) = 0.30, *p* < 0.001; total score and experience, *r* (182) = 0.29, *p* < 0.001; and total score and oncology experience, *r* (182) = 0.24, *p* = 001.

## Discussion

This study explored nurses’ knowledge of neutropenia in cancer patients. It also underscores the association of nurses’ knowledge with their demographic variables. A comprehensive literature search revealed only a few studies that measured nurses’ knowledge of CIN (Naghdi et al. 2019; Nirenberg et al. 2010; Tarakcioglu Celik and Korkmaz 2017; Teleb Osman and Mohamed Bayoumy 2016), hence limiting points of direct comparison with the present study. Relevant studies pertinent to nurses’ knowledge of other aspects of oncology care and infection control were included to enrich the discussion.

The findings show that nurses have moderate knowledge of neutropenia (mean score = 16.3 / 30, SD = 3.7). This is similar to the findings of some studies (Naghdi et al. 2019; Teleb Osman and Mohamed Bayoumy 2016), but lower than other studies which yielded a high knowledge level (Nirenberg et al. 2010; Tarakcioglu Celik and Korkmaz 2017). Out of the 30 items, respondents scored poorly on 14, with less than 50% giving the correct answer. These low-scoring items can be classified into four categories: the definition and criteria for CIN; identification and monitoring of signs and symptoms of CIN as well as infection among CIN patients; general nursing care of CIN patients (vital signs monitoring, hygiene, diet); and infection-prevention mechanisms (wearing of personal protective equipment (PPE), isolation protocol). Disconcertingly, the majority of the nurses failed to identify the basic definition of neutropenia, and this item yielded the lowest score when compared to other studies (Naghdi et al. 2019; Tarakcioglu Celik and Korkmaz 2017). Although the majority of the nurses were not able to identify that neutrophil should be below 1500 cells/mm^3^, their score was encouraging compared to one study (Naghdi et al. 2019). Nurses scored lowest on the frequency of rinsing neutropenic patients’ mouths, similar to the findings of one study (Naghdi et al. 2019), but higher than in another (Tarakcioglu Celik and Korkmaz 2017). The second-lowest item was about frequency of bathing CIN patients, contrary to the results of other studies which yielded higher scores (Naghdi et al. 2019; Tarakcioglu Celik and Korkmaz 2017). The third lowest item was about the use of PPE in neutropenic patient care: higher than in one study (Naghdi et al. 2019) but far lower than in another study (Tarakcioglu Celik and Korkmaz 2017). Differences in the level of nurses’ knowledge across studies can be attributed to various reasons such as study settings and respondents’ characteristics. Some studies collected data from hospitals with rigorous neutropenic patient care protocol, or where the majority of respondents working in specialized oncology units had advanced oncology certification or had received in-service education on CIN and infection control.

The result of the study in terms of nurses’ level of knowledge is generally unfavorable and requires prompt attention and action. Remarkably, patients with cancer recover faster, feel safer and are more secure when they are cared for by nurses who are knowledgeable and equipped with competent skills (Corner et al. 2013; Kvåle and Bondevik 2010). Also, higher knowledge is associated with a higher level of practice (Naghdi et al. 2019). Strategies to improve nurses’ knowledge include the provision of courses or advanced certification, professional training and workshops, orientation training at the beginning of employment, simultaneous cutting-edge theoretical and practical programs to bridge the theory–practice gap, and improving nurses’ attitude toward infection-control principles (Nasiri et al. 2019). The use of oncology simulation with the effective integration of mnemonics, road maps and case-based learning also improved nurses’ knowledge, perceived competence and skills acquisition (Linnard-Palmer 2012). The intensive implementation of evidence-based nursing protocols which consisted of lecture, demonstration, re-demonstration and distribution of printed protocol booklets have drastically improved their knowledge and practice in caring for patients with CIN (Teleb Osman and Mohamed Bayoumy 2016). Moreover, ensuring the availability and accessibility of CIN clinical practice guidelines encourage better implementation at the bedside (Nirenberg et al. 2010). Nurses must also update themselves with the latest research evidence on CIN care to guide their practice (Kaplow and Spinks 2015). Finally, integrating oncology nursing courses in nursing curricula is strongly recommended.

Significant associations between nurses’ knowledge of CIN and their socio-demographic variables were also established in the study. The findings reveal a positive association with their nursing degree, with those holding a post-graduate degree being more likely to have greater knowledge than their BSN counterparts, as in other studies (Alojaimy et al. 2021; Nirenberg et al. 2010; Sharour 2019; Suliman et al. 2018; van Veen et al. 2017). Also, nurses who had received an educational course on oncology or CIN demonstrated higher knowledge scores than those who had not, contrary to other findings (Suliman et al. 2018). Evidence indicates that nurses with higher education, training or advanced certification exhibit higher levels of competence, confidence and compliance with guidelines (Al-Rawajfah et al. 2013; Nirenberg et al. 2010). Hence, it is essential that nurses pursue professional development in terms of post-graduate studies, advanced oncology certification, and participation in training and workshops related to oncology nursing or CIN. Hospital and nursing administrators must look into these more intently by providing scholarship grants, more flexible working hours, and equal opportunity for nurses to enrich themselves professionally.

Nurses working in oncology units had a significantly higher knowledge score than nurses working in other units, in contrast with other findings (Sarani et al. 2016). The former concentrate on the care of cancer patients on a daily basis, while those in other units may receive them less frequently. Because the oncology unit is highly specialized, they have more opportunities to reinforce their knowledge throughout their clinical practice. Filipino nurses had a significantly higher knowledge score than other nationalities; this may be attributed to the differences in undergraduate preparation in their respective countries. Older nurses are more likely to possess greater knowledge of CIN, similar to other studies (Hafeez et al. 2020; van Veen et al. 2017), but this may be indirectly linked to their years of clinical practice experience. Nurses with more years of general nursing experience as well as oncology experience had higher knowledge scores, similar to other studies (Hafeez et al. 2020; Teleb Osman and Mohamed Bayoumy 2016). As nurses age and mature in their profession, their cumulative clinical experience serves as an opportunity to develop their knowledge in neutropenic patient care.

These findings call for the necessity of specialization in oncology nursing. This can be achieved by ensuring that core nurses for oncology care remain in the same unit for a significant period without interruption, to allow for repeated exposure to similar cases, hence making the clinical environment a significant learning ground to enrich their knowledge and caliber in caring for patients with CIN.

### Implications for nursing practice

Chemotherapy-induced neutropenia is associated with various serious consequences such as life-threatening infection, delays in cancer treatment, higher morbidity and mortality, and excessive healthcare cost (Abou Saleh et al. 2013; Crawford et al. 2004; Lyman et al. 2010; Schelenz et al. 2012). Sensible strategies to augment nurses’ knowledge of CIN are well-established, such as pursuing higher studies, provision of certification, courses, training, and workshops (Nasiri et al. 2019); the use of simulation (Linnard-Palmer 2012); making CIN clinical practice guidelines readily available (Nirenberg et al. 2010); and radical and intentional implementation of evidence-based practice (Kaplow and Spinks 2015). Fundamentally, nurses must reconsider their core values as the heritage and bloodline of their profession. They must assume responsibility and critically analyze their own level of knowledge and practice, exercise leadership and strengthen their advocacy (Challinor et al. 2020; Nirenberg et al. 2010). The empathetic use of self, a caring attitude, and genuine concern must motivate them to refine their knowledge and patient care (Fall-Dickson and Rose 1999).

### Limitations

This cross-sectional study was conducted in a single tertiary hospital in Oman. Keeping this in mind, readers must exercise caution in interpreting the generalizability of study findings. Self-reported questionnaires as data collection also have the risk of response bias, and using an observational approached may yield more reliable data. Lastly, the dearth of available literature on the topic limited points for a comprehensive comparison. This highlights the need for future studies to explore CIN knowledge among nurses, as well as other variables such as attitude, compliance, practices, and interventional studies to enrich nursing scholarship and evidence-based practice.

## Conclusion

The results of the current study showed that nurses have a moderate level of knowledge of CIN. The findings call for the need for further education and training. As a long-term plan, this might be accomplished by encouraging nurses to pursue post-graduate education or oncology-specialized certification, supported by scholarship grants. However, deliberate plans for short courses, training and workshops on oncology or CIN would have a more immediate impact on nurses’ knowledge and clinical practice. Finally, integrating oncology nursing education within nursing curricula is needed.

## Data Availability

The datasets analysed during the current study are available from the first author [Mohammad Al Qadire] on reasonable request.
